# Development of an AI-Supported Clinical Tool for Assessing Mandibular Third Molar Tooth Extraction Difficulty Using Panoramic Radiographs and YOLO11 Sub-Models

**DOI:** 10.3390/diagnostics15040462

**Published:** 2025-02-13

**Authors:** Serap Akdoğan, Muhammet Üsame Öziç, Melek Tassoker

**Affiliations:** 1 Department of Biomedical Engineering, Faculty of Technology, Pamukkale University, Denizli 20160, Türkiye; sakdogan18@posta.pau.edu.tr; 2 Department of Oral and Maxillofacial Radiology, Faculty of Dentistry, Necmettin Erbakan University, Konya 42090, Türkiye; dishekmelek@gmail.com

**Keywords:** mandibular third molar extraction, oral surgery, panoramic radiography, Pederson difficulty index, YOLO11

## Abstract

**Background/Objective:** This study aimed to develop an AI-supported clinical tool to evaluate the difficulty of mandibular third molar extractions based on panoramic radiographs. **Methods:** A dataset of 2000 panoramic radiographs collected between 2023 and 2024 was annotated by an oral radiologist using bounding boxes. YOLO11 sub-models were trained and tested for three basic scenarios according to the Pederson Index criteria, taking into account Winter (angulation) and Pell and Gregory (ramus relationship and depth). For each scenario, the YOLO11 sub-models were trained using 80% of the data for training, 10% for validation, and 10% for testing. Model performance was assessed using precision, recall, F1 score, and mean Average Precision (mAP) metrics, and different graphs. **Results:** YOLO11 sub-models (nano, small, medium, large, extra-large) showed high accuracy and similar behavior in all scenarios. For the calculation of the Pederson index, nano for Winter (average training mAP@0.50 = 0.963; testing mAP@0.50 = 0.975), nano for class (average training mAP@0.50 = 0.979; testing mAP@0.50 = 0.965), and medium for level (average training mAP@0.50 = 0.977; testing mAP@0.50 = 0.989) from the Pell and Gregory categories were selected as optimal sub-models. Three scenarios were run consecutively on panoramic images, and slightly difficult, moderately difficult, and very difficult Pederson indexes were obtained according to the scores. The results were evaluated by an oral radiologist, and the AI system performed successfully in terms of Pederson index determination with 97.00% precision, 94.55% recall, and 95.76% F1 score. **Conclusions:** The YOLO11-supported clinical tool demonstrated high accuracy and reliability in assessing mandibular third molar extraction difficulty on panoramic radiographs. These models were integrated into a GUI for clinical use, offering dentists a simple tool for estimating extraction difficulty, and improving decision-making and patient management.

## 1. Introduction

The frequency of impacted mandibular molars is reported in the literature to be between 16.7% and 68.6% [1]. The surgical extraction of impacted mandibular third molars is one of the most frequently performed and challenging procedures in oral and maxillofacial surgery. Proper evaluation of the procedure’s difficulty before surgery is crucial to estimating postoperative risks, such as swelling, pain, limited mouth opening, dry socket, and inferior alveolar nerve injury. Additionally, it is essential to predict the operation’s duration and determine the time the patient should allocate for the appointment [1,2]. The difficulty of extracting impacted third molars varies significantly between cases. In some instances, the procedure can be relatively straightforward, involving the removal of alveolar bone and tooth separation. However, extraction may require general anesthesia in more complex cases where the molar is deeply embedded [2]. Therefore, the operating oral surgeon needs scientific evidence regarding each case’s estimated surgical difficulty level [1,3]. This way, the patient will be better informed and mentally prepared before surgery, and the physician can obtain appropriate informed consent regarding the clinical scenario [4]. Several indices have been suggested in the literature to evaluate the extraction difficulty of impacted mandibular third molars preoperatively [4]. The most widely used of these, the Pederson index [5], is derived from the Winter and Pell and Gregory classifications [2,6,7]. This evaluates and scores third molar extractions based on radiographic factors, including the tooth’s position, depth, and its relationship to the mandibular ramus [1]. An important limitation of the Pederson index is that it does not include clinical judgment. However, due to its high specificity, the index is reported to be effective in efficiently planning surgeries by eliminating cases that are unlikely to present significant difficulties [4]. Panoramic radiographs (PRs), which are routinely taken in the clinic and provide a single view of the entire jaw structure, provide a clear view for positioning the mandibular third molars. However, for detailed analysis of the molars, such as their relationship with the inferior alveolar canal, cone beam computed tomography (CBCT) images may provide higher diagnostic accuracy [8].

Over the last decade, advancements in artificial intelligence (AI) and its integration into medicine and dentistry have led to a rapid increase in research on deep learning-based models for AI-assisted medical diagnosis [9,10,11]. Studies that were carried out with conventional machine learning for a long time have been replaced by deep learning models because deep learning models automatically extract and select features across layers. Many feature extraction and selection methods had to be tried in traditional machine learning methods, and the best methodology was determined after many trials. Due to these advantages, many deep learning models have been proposed in recent years [12,13]. Deep learning studies focused on mandibular third molars in PRs have included the determination of the eruption potential of mandibular third molars [14], wisdom tooth detection [9,15,16], segmentation [17,18], age estimation [19], estimation of the time required for mandibular third molar extraction [20], classification of the developmental stages of third molars in a fully automated way [21], determination of the anatomical relationship of mandibular third molars to the mandibular canal [16,22,23,24,25,26,27,28], classification of mandibular third molars with different approaches [24], prediction of Pell and Gregory classes [29,30,31], determination of Winter angulation [9,15,29,30,31,32], and prediction of extraction difficulty according to indexes [33,34,35,36,37]. The variety of studies conducted on mandibular third molars clearly demonstrates the clinical importance of these teeth. AI-based studies have provided significant advances in many areas such as the detection, classification, segmentation, determination of anatomical relationships, and prediction of the extraction difficulty of these teeth. However, despite all these developments, manual evaluation of extraction difficulty is still a common practice [38,39]. These traditional approaches are time-consuming and prone to human error. Conversely, studies evaluating extraction difficulty with AI-based methods in PRs are quite limited and their current accuracy rates are still insufficient. This study aimed to automatically detect, classify, and score the extraction difficulty of impacted mandibular third molars based on the Pederson index on panoramic radiographs using YOLO11 state-of-the-art models. YOLO11, the latest version of the YOLO (You Only Look Once) family, which is popularly used in the literature for object detection, segmentation, pose estimation, object tracking, and classification, was introduced in October 2024. This model has increased feature extraction capabilities with improved spine and neck architectures and provides higher efficiency, speed, and accuracy values compared to previous versions and competing models. YOLO11, which can adapt to the environment in difficult tasks, offers a practical use to researchers with its user-friendly interface and framework provided by the Ultralytics library (https://github.com/ultralytics accessed on 1 December 2024). Because of its efficiency and ease of integration into different systems, YOLO11 is expected to have many future applications across various industrial sectors, including healthcare [40]. Many applications to be performed with this algorithm will take their place in medicine to reduce complications that inexperienced general practitioners may encounter, increase the patient’s postoperative comfort, and eliminate the need for a jaw surgeon’s expertise when necessary. At the end of this study, the tooth extraction difficulties obtained with YOLO11 are automatically printed on the images, a graphical user interface (GUI) is designed for clinical use, and the results are discussed.

## 2. Materials and Methods

### 2.1. Data Collection and Image Preprocessing

This study was performed in accordance with the Declaration of Helsinki and protocol number 2024/424 obtained from the Ethics Committee of Necmettin Erbakan University Faculty of Dentistry, Department of Oral and Maxillofacial Radiology, on 25 April 2024. A total of 2000 PRs of mandibular third molars were collected from patients who visited the Faculty of Dentistry for dental check-ups between 2023 and 2024. The dataset was obtained from two different devices: NewTom GiANO HR (Verona, Italy) and 2D Veraviewpocs (J MORITA MFG corp, Kyoto, Japan). Clear visualizations of the root structure, tooth positioning, and bone level of the third molars were established as the inclusion criteria, while cases involving complete extractions, surgical interventions, trauma, cystic lesions, tumors, or severe maxillofacial deformities were designated as exclusion criteria. All images were converted to Portable Network Graphics (PNG) format with 8-bit depth and resized to 1024 × 532 using the bilinear interpolation method.

### 2.2. Radiological Assessment and Image Annotation

Radiologic evaluations were performed according to the Pederson index [5], which is widely used in the literature to predict the difficulty of extraction of mandibular third molars. This index divides the spatial position and depth of the third molars into 3 subcategories according to the Pell and Gregory system [6] and the angulation into 4 subcategories according to the Winter classification system [7]. Thus, 36 different possibilities were calculated (Table 1) and extraction difficulty was slightly difficult, moderately difficult, and very difficult. According to the Pell and Gregory and Winter classification systems, the following criteria were used for the radiologic evaluation of PRs. Table 1 shows the scores for each subcategory and total score values.

**Angulation (Winter)** [7]:

**Vertical:** There is an angle between 10° and −10° between the long axis of the mandibular third molar and the long axis of the second molar, and the tooth is in a vertical position.

**Mesioangular:** There is an angle between 11° and 79° between the long axis of the mandibular third molar and the long axis of the second molar, and the crown of the tooth is inclined towards the second molar.

**Horizontal:** There is an angle between 80° and 100° between the long axis of the mandibular third molar and the long axis of the second molar, and the tooth is in a horizontal position.

**Distoangular:** There is an angle between −11° and −79° between the long axis of the mandibular third molar and the long axis of the second molar and the crown of the tooth is inclined towards the back of the jaw.

**Ramus Relation (Pell and Gregory):** Position of the third molar relative to the mandibular ramus [6].

**Class I:** The space between the mandibular ramus and the distal root of the second molar is sufficient for the mesiodistal diameter of the third molar to be located.

**Class II:** The space between the mandibular ramus and the distal root of the second molar is insufficient for the mesiodistal diameter of the third molar to be located.

**Class III:** The third molar tooth is completely located in the mandibular ramus.

**Depth (Pell and Gregory):** Depth level according to the occlusal plane [6].

**Level A:** The uppermost part of the third molar (occlusal surface) is at or above the occlusal plane of the adjacent second molar.

**Level B:** The uppermost part of the third molar is between the cementoenamel junction of the second molar and the occlusal plane.

**Level C:** The uppermost part of the third molar is located apical to the cementoenamel junction of the adjacent second molar.

**Figure 1:**
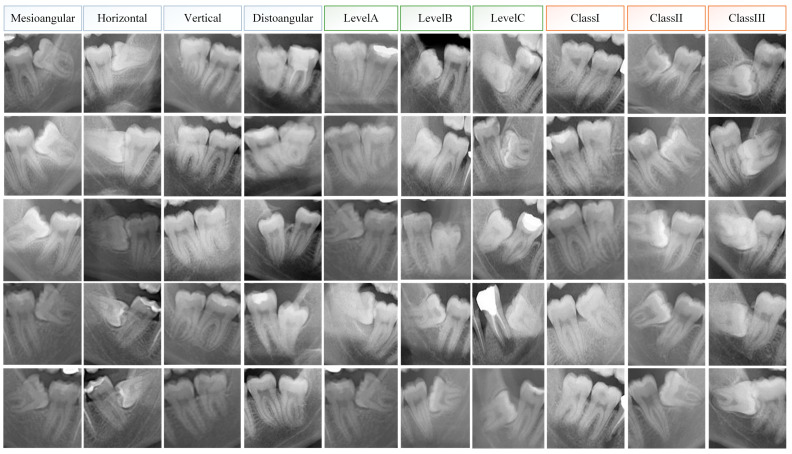
Radiological evaluation of mandibular third molar PR patches based on Pell and Gregory classification and Winter’s angulation.

**Figure 2:**
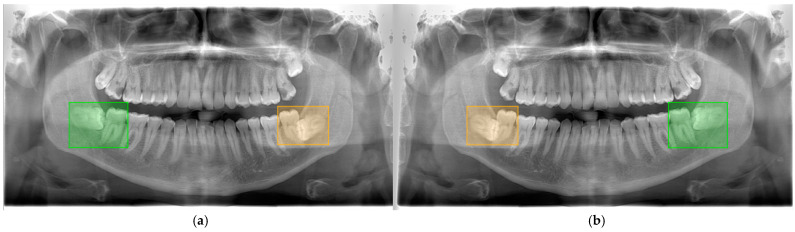
(**a**) Original annotated PR. (**b**) Horizontally flipped and annotated PR.

Considering these criteria, all PRs were examined by an oral radiologist (MT) with 13 years of experience and prepared for this study. Figure 1 shows the image patches prepared according to the relevant criteria from the dataset and the class information is specified. As seen in Figure 1, since the dental anatomical structures of individuals are different, each patch shows a unique pattern. From the 2000 collected PRs, 1400 datasets were created separately for class, level, and angulation. Since each scenario needs to be evaluated independently, a separate AI model should be created for each. The PRs were doubled by horizontally flipping them. Therefore, a total of 8400 PRs were created from 2800 data for each scenario. PRs were annotated using the bounding box with the browser-based labeling tool makesense (https://www.makesense.ai/ accessed on 1 December 2024). In the annotation performed by MT, the inclusion of the third and second molar teeth with their roots in the bounding box was taken into account as the labeling criterion, and a total of 8400 PR images were annotated. Figure 2 shows how an original and a horizontally flipped image were annotated.

### 2.3. YOLO11 Sub-Models, Transfer Learning and Fine-Tuning

In this study, the n (nano), s (small), m (medium), l (large), and x (extra-large) sub-models of the YOLO11 algorithm were trained and tested for three different scenarios. The library released by Ultralytics was preferred because it offers user-friendly Python scripts and cloud-based training support. Training and testing operations were performed on TESLA A100 GPUs using Google COLAB Pro+. Data were divided into 80% training, 10% validation, and 10% test sets for each scenario. Data augmentation was performed after the data were separated to ensure that horizontally flipped versions of the original images were not present in other files. Since the current YOLO models were trained with the COCO dataset, they can recognize 80 objects. However, to train with specialized datasets, the capabilities of existing networks can be transferred to the new model using transfer learning operations. Among the hyperparameters of these models, the epoch count was set to 600 and the mini-batch size was set to 8, while the other parameters were left at their default values (optimizer: SGD (lr = 0.01, momentum = 0.9), patience: 100). Due to the structure of the model, the following mosaic data augmentation operations were applied before the images entered the network (Blur (*p* = 0.01, blur_limit = (3, 7)), MedianBlur (*p* = 0.01, blur_limit = (3, 7)), ToGray (*p* = 0.01, num_output_channels = 3, method = ‘weighted_average’), and CLAHE (*p* = 0.01, clip_limit = (1.0, 4.0), tile_grid_size = (8, 8)). So, the generalization ability of the model was increased by using the mosaic data augmentation technique. As a result of the training, artificial intelligence files with the .pt extension were produced by the framework. Test data that the system had not seen before were passed through these models, and the test performance was evaluated separately for three scenarios.

### 2.4. Performance Evaluation Metrics

The performance of the training and testing processes was evaluated separately for each scenario. The Ultralytics framework automatically calculates the performance metrics. The system first calculates TP (True Positive), which indicates instances that the model predicts as positive, FP (False Positive), which indicates instances that the model predicts incorrectly, and FN (False Negative), which indicates instances that the model fails to predict or does not find an essentially positive instance. Based on these values, precision (P), which indicates the ratio of TP predictions to total positive predictions (Equation (1)), and recall (R), which demonstrates the ratio of TP predictions to total TPs and FNs (Equation (2)), are calculated, and the F1 score (F1-S) is acquired with the harmonic average of these two metrics (Equation (3)). Average Precision (AP) is used to measure the overall performance of the model at different threshold values (Equation (4)) (k: number of classes). The mean Average Precision (mAP) value is then calculated by taking the average AP across all classes (Equation (5)). In particular, mAP@0.5 and mAP@0.5:0.95 metrics are calculated by taking the average of the 50% IoU (Intersection over Union) threshold and IoU thresholds ranging from 50% to 95%, respectively. These metrics provide a detailed analysis of the performance of an object detection model. The results of this study are supported with graphs showing the performances according to the number of epochs, confusion matrices, and precision–recall graphs.

(1) $$precision=\frac{TP}{TP+FP}$$

(2) $$recall=\frac{TP}{TP+FN}$$

(3) $$F1-score=2\times \frac{Precision\times Recall}{Precision+Recall}$$

(4) $$AP_{k}=\int_{0}^{1}P_{k}(R_{k})dR_{k}$$

(5) $$mAP=\frac{1}{k}\sum_{i=1}^{k}AP_{i}$$

## 3. Results

### 3.1. Training and Testing Performance Results

Evaluating the performance of the models in both the training and testing phases provides important information about the generalization ability and correct detection ability regarding the detection and classification of third molars under three different scenarios. In this section, the performances will be examined according to the basic performance measurement metrics of precision, recall, F1 score, mAP, precision–recall, confusion matrix (CM), and loss graphs obtained during the training and testing processes. Table 2 shows the training and testing results for Winter angulation (Img: images; Ins: instances). As shown in Table 2, the performance values of the YOLO11 models are close to each other, but they appear to maintain a good balance between precision and recall, particularly in the Distoangular class. In the “Mesioangular” and “Horizontal” classes, all models show strong results, while in the “Distoangular” and “Vertical” classes, the accuracies are relatively lower. The nano model, with its lightweight structure, provides a balanced performance with high generalization capability, and has more stable results than other models, especially in the “Distoangular” class. Due to these advantages, the YOLO11n model (average testing mAP@0.5 = 0.975, mAP@0.5:0.95 = 0.849) was selected for Winter classification in extraction difficulty index calculations.

**Figure 3:**
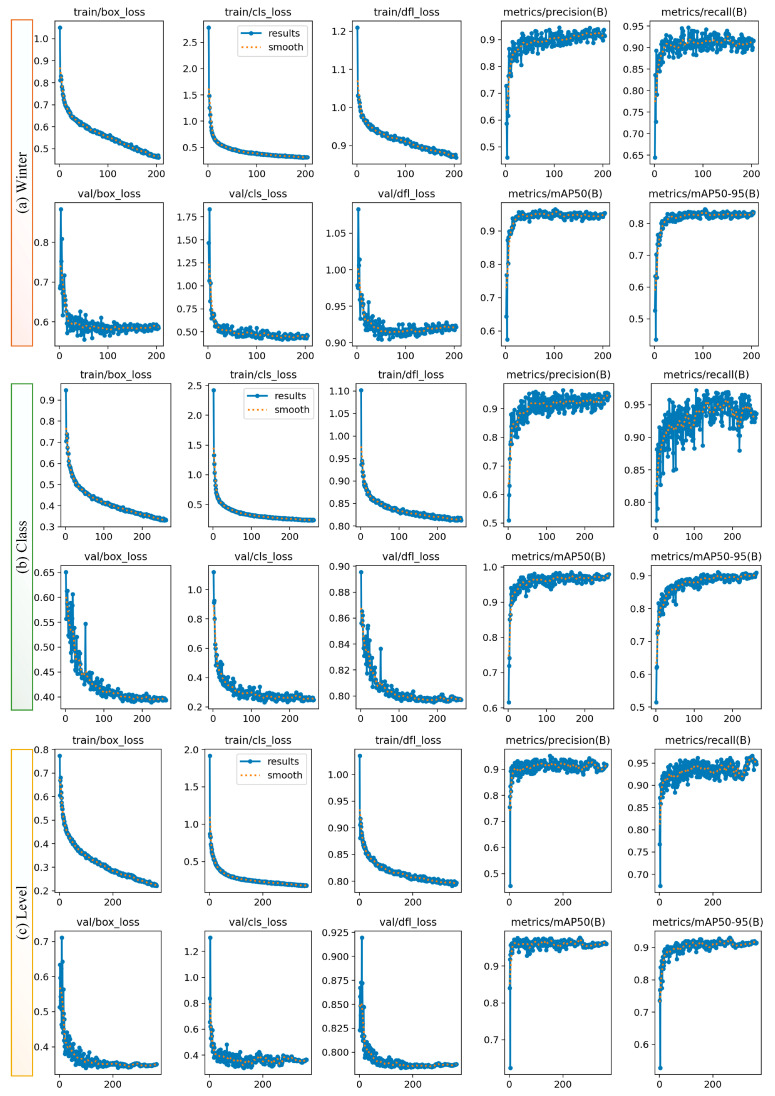
Graphs showing the loss values (box_loss, cls_loss, dfl_loss) and performance metrics (precision, recall, mAP50, mAP50–95) of the model during the training and validation processes across epochs: (**a**) Winter for YOLO11n, (**b**) class for YOLO11n, (**c**) level for YOLO11m.

**Figure 4:**
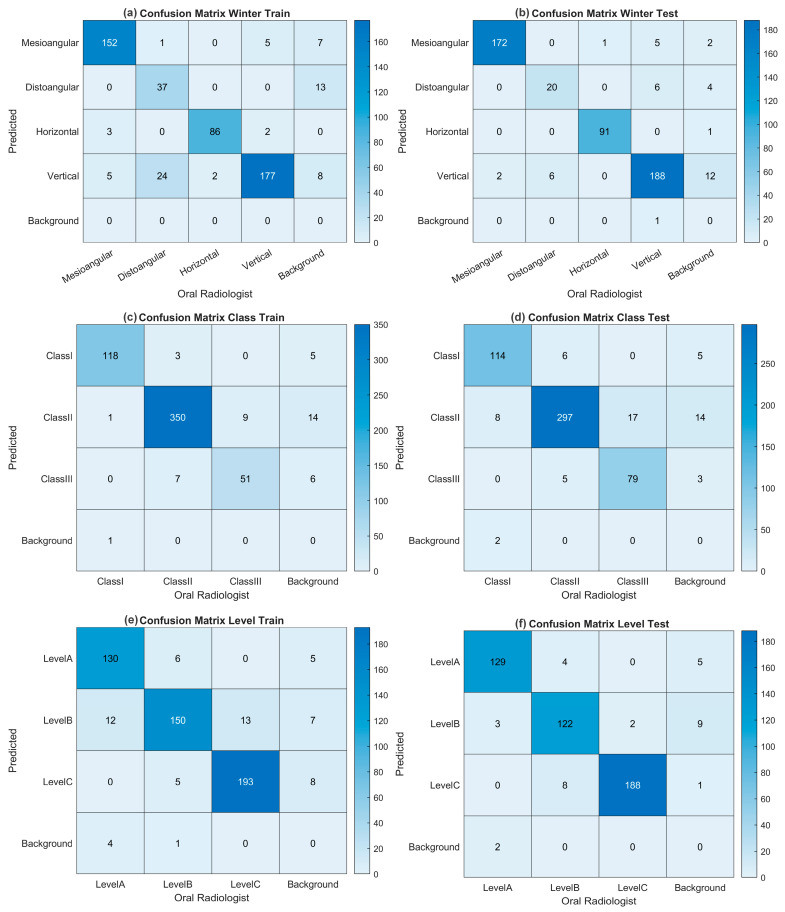
(**a**) Training CM for Winter angulation. (**b**) Testing CM for Winter angulation. (**c**) Training CM for class. (**d**) Testing CM for class. (**e**) Training CM for level. (**f**) Testing CM for level.

**Figure 5:**
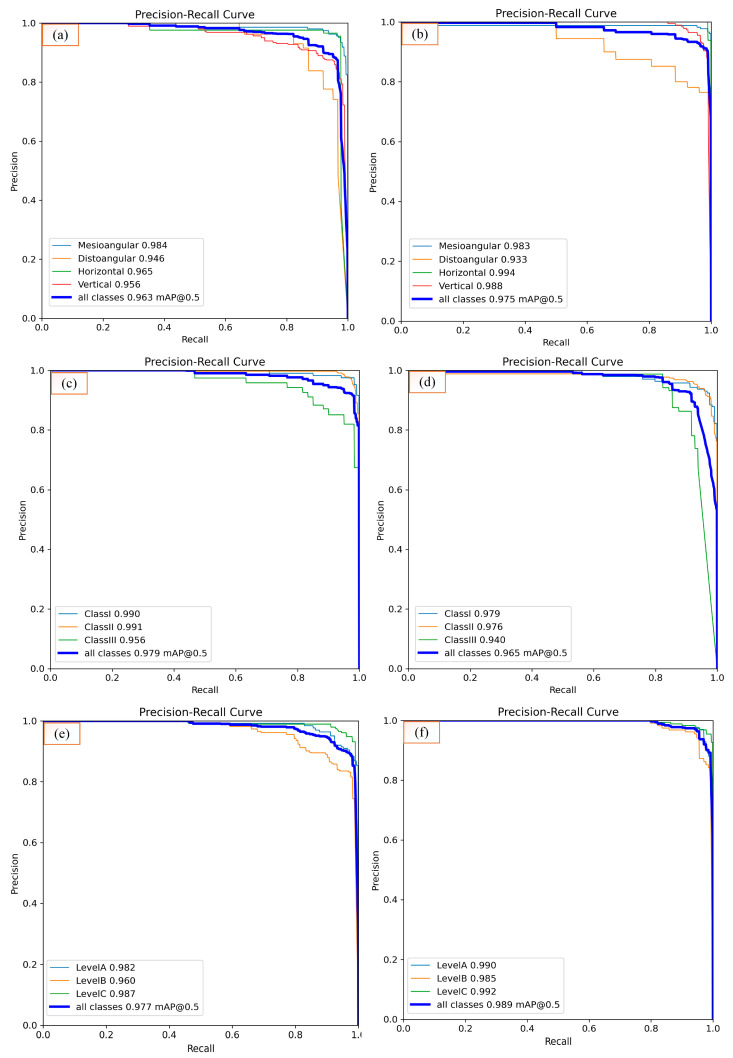
(**a**) Training precision–recall curve for Winter angulation, (**b**) testing precision–recall curve for Winter angulation, (**c**) training precision–recall curve for class, (**d**) testing precision–recall curve for class, (**e**) training precision–recall curve for level, (**f**) testing precision–recall curve for level.

Table 3 shows that Class I and Class II models achieve high precision and recall values, with their performance outputs exhibiting similar behavior. In Class III, strong performance is observed despite the relatively low data volume, but its performance is relatively low compared to the other classes. The YOLO11n model provides similar accuracy metrics compared to larger models while offering the advantage of lower computational cost and faster processing. The YOLO11n model (average testing mAP@0.5 = 0.965; mAP@0.5:0.95 = 0.886) was used to determine the extraction difficulty index because of its lightweight structure and its effectiveness, especially in dense sample groups such as Class I and II. In general, all models demonstrated consistency at Levels A, B, and C, achieving high precision and recall values. In particular, the larger models (large and extra-large) achieved high accuracy values at all levels, while the smaller models (nano and small) achieved sufficient accuracy with low computational cost. The YOLO11 medium model stands out with a balanced performance at all levels and reaches a high value of 0.949 mAP@0.5:0.95 on average, especially on the test data. The YOLO11m model was preferred for the determination of extraction difficulty because it is advantageous in terms of computational cost compared to larger models and has the highest test mAP@0.5:0.95. When all the results were evaluated, it was decided to use YOLO11n for Winter angulation (average testing mAP@0.5 = 0.975; mAP@0.5:0.95 = 0.849), YOLO11n for class (average testing mAP@0.5 = 0.965; mAP@0.5:0.95 = 0.886), and YOLO11m for level (average testing mAP@0.5 = 0.989; mAP@0.5:0.95 = 0.949) in GUI design and determination of the tooth extraction difficulty index. Figure 3 graphically shows the performance of these three selected models at the time of training and validation. The fact that the training and validation losses (box_loss, cls_loss, and dfl_loss) decreased rapidly in all tasks and stabilized before the completion of 600 epochs with the early stopping operation reveals that the model performs strong learning in both location estimation and classification. Precision and recall values above 0.9 indicate that the model performs with high accuracy and low error rate, while mAP@0.5 values above 0.9 and mAP@0.5:0.95 values above 0.8 in Winter, class, and level tasks prove that the models perform consistently and successfully at IoU thresholds. Moreover, the validation losses are in line with the training losses, indicating that there are no signs of overlearning and that the model has a high generalization capacity across different datasets. For the nano model, training in the class scenario lasted 1 h and 57 min, with early stopping applied at the 262nd epoch, while in the Winter scenario, training lasted 1 h and 31 min, with early stopping occurring at the 204th epoch; for the medium model in the level scenario, training lasted 3 h and 25 min, with early stopping applied at the 363th epoch. While 5.5 MB AI files with the *.pt extension were obtained for nano models, 40.5 MB AI files were obtained for the medium model. For the selected models, training and testing confusion matrices for level, class, and Winter are given in Figure 4, and precision-recall plots are shown in Figure 5.

### 3.2. Visual Results

**Figure 6:**
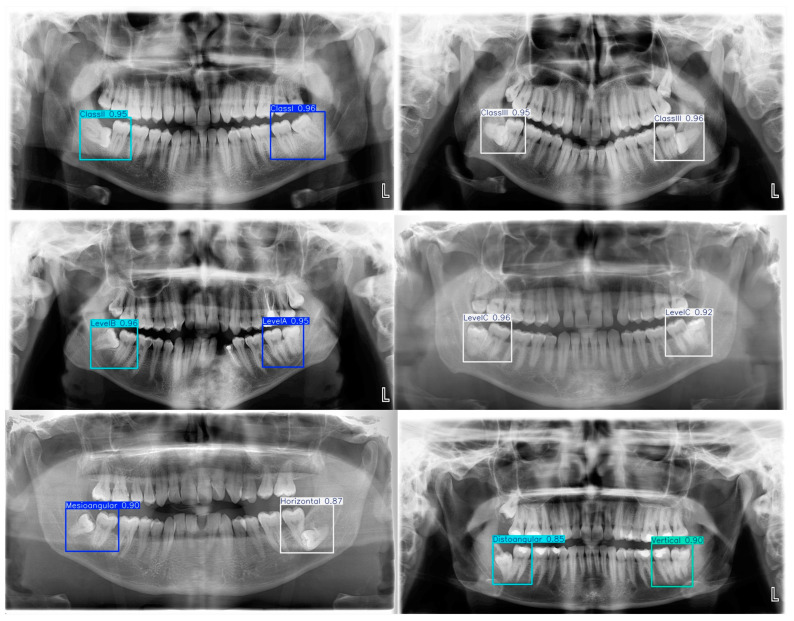
Winter angulation and Pell and Gregory class prediction on six test images using YOLO11 weights. ‘L’ is the left side of the patient.

The prediction results in the test images created for Winter angulation, class, and level were examined by an oral radiologist. It was observed that the second and third molars were correctly boxed in all images, accurately detecting the corresponding localizations. Only some class information was occasionally mispredicted in relation to each other. Since each PR image is personalized and has different anatomical structures, it was observed that the YOLO11 model gave a high success rate in localizing the relevant region. Figure 6 shows six test images with automatic localization and class prediction.

### 3.3. Calculation of Pederson Index and Assistant GUI Tool

The Pederson index, a scoring system used to assess the difficulty level of third molar extractions, is calculated based on the tooth’s position relative to the occlusal plane, angulation, and the relationship of the tooth to the mandibular ramus. Angulation is scored as mesioangular (1), vertical (3), horizontal (2), and distoangular (4), with the relationship to the ramus denoted as Class I (1) Class II (2), and Class III (3), and depth denoted as Level A (1), Level B (2), and Level C (3). According to the combination of conditions in the tooth, the values obtained as a result of the total are 3–4 points: slightly difficult; 5–6 points: moderately difficult; 7–10 points: very difficult. Table 4 shows all possible combinations (36 different combinations) and total scores based on angulation, ramus relationship, and depth.

**Figure 7:**
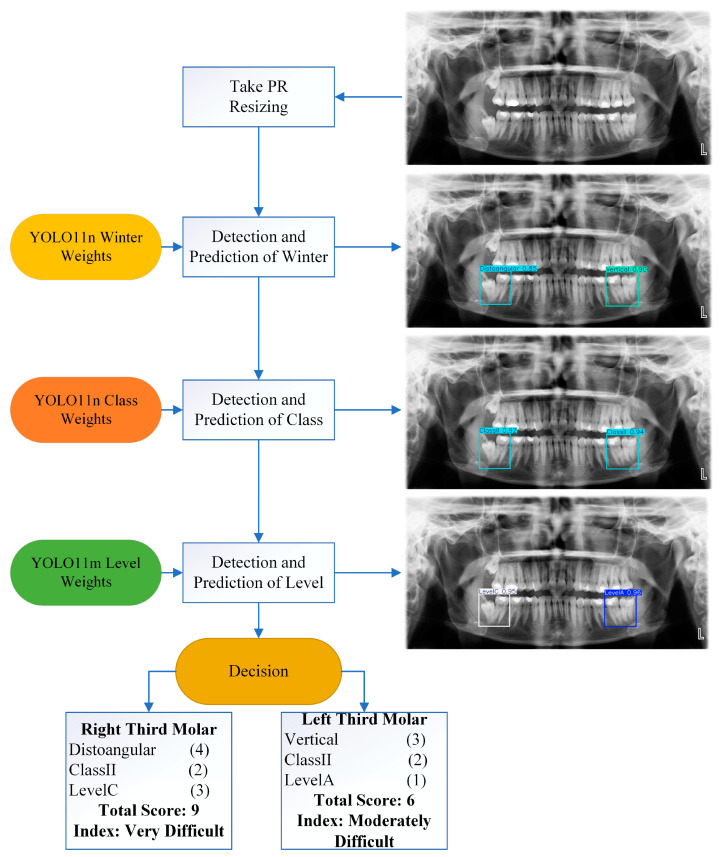
Flow diagram of the system designed for the automatic determination of the mandibular third molar extraction difficulty index.

Figure 7 shows the pipeline designed in this study to calculate the extraction difficulty for a single tooth. The system takes an image and first converts it to 8-bit depth and then rescales it to 1024 × 532. The image is sent to a YOLO11n model trained for angulation prediction, a YOLO11n model trained for class prediction, and a YOLO11m model trained for level prediction, respectively. Each model stores both the prediction results and class localization in a variable and can provide visualization outputs. This process is performed separately for the left and right third molars to determine the extraction difficulty score and grade.

**Figure 8:**
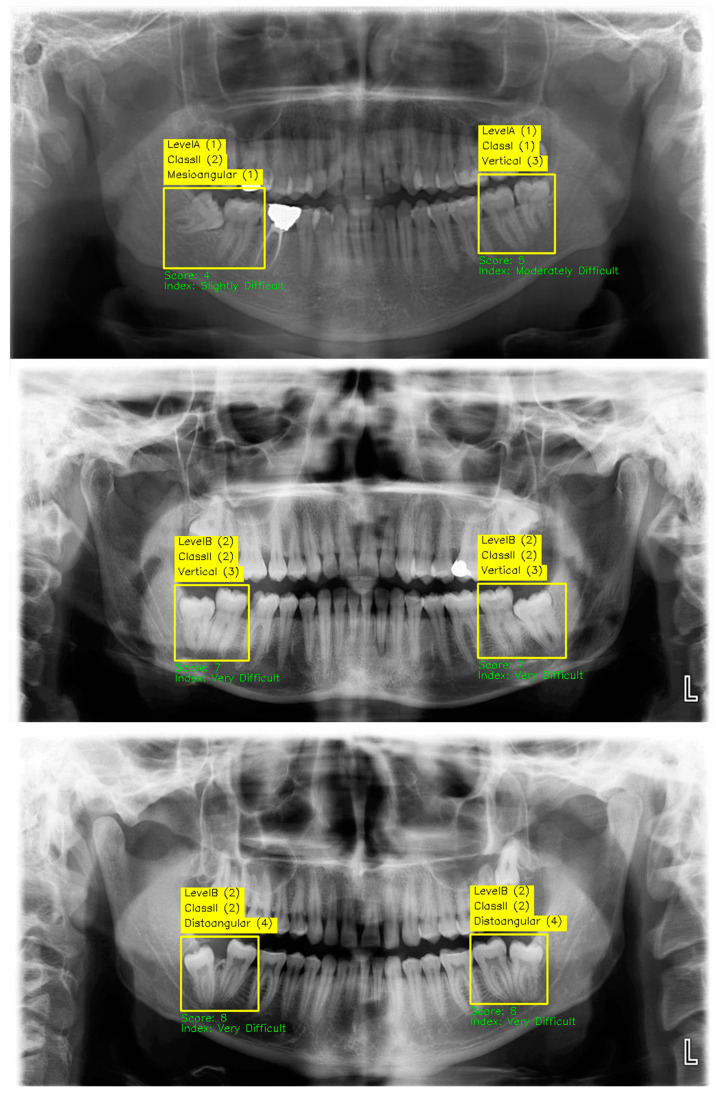
Analysis of 3 test images in terms of the Pederson index using the pipeline developed in Figure 7.

**Figure 9:**
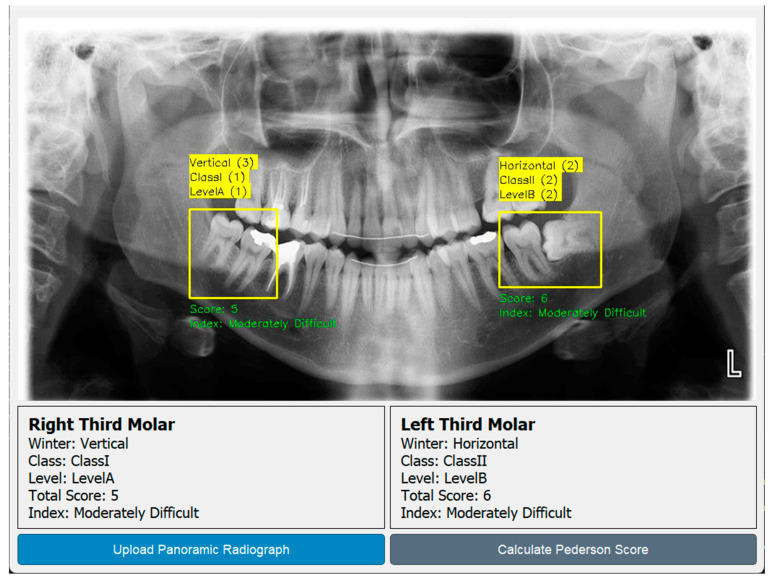
GUI developed for automatic calculation of Pederson index.

Using the pipeline developed in Figure 7, the prediction and scoring images of three test images are given in Figure 8. The pipeline designed in Figure 7 was developed as a Python script and embedded in a GUI, as seen in Figure 9. In the GUI designed using the PyQT library, all analysis can be performed with a single button. Thus, dentists will be able to calculate the third molar extraction difficulty score without being exposed to complex coding processes.

In the experiments conducted to calculate the Pederson index of a total of 514 mandibular third molars on 300 test images performed using the GUI, the model detected 507 bounding boxes. Of these detected boxes, 12 were evaluated as FP, and 7 were not detected at all and were included in the FN category. However, out of the 24 boxes for which the Pederson index could not be calculated (N/A), 3 were added to FP due to over-detection, and 21 were added to FN due to under-detection. As a result of these adjustments, the total number of FPs was determined as 15, the total number of FNs was 28, and the total number of TPs was determined as 486. When the model’s performance was examined, precision was calculated as 97.00%, recall as 94.55%, and F1 score as 95.76%. These results demonstrate that the proposed method based on YOLO11 offers high accuracy and reliability in the detection of mandibular third molars and the calculating of the Pederson index.

## 4. Discussion

This study developed an AI-assisted clinical tool employing YOLO11 models to predict the difficulty of mandibular third molar extractions, utilizing the widely accepted and validated [44] Pederson difficulty index criteria. As a result of the comparison of YOLO11 sub-models, the nano model for class, the medium model for level in the Pell and Gregory category, and the nano model in the Winter category were preferred for index calculation. It became evident that the sub-models generally yielded similar results to each other, and high results were obtained especially in terms of mAP@50 (mean Average Precision at IoU threshold 0.5) values. High values of mAP@50 indicate that the model performs well, detects the correct objects in positive predictions in the Pell and Gregory and Winter categories, and matches them with a high confidence score. YOLO11 weights were applied sequentially for class, level, and Winter on a PR image and Pederson difficulty index scores were automatically determined. In the evaluation performed by an oral radiologist on 150 test images, the proposed pipeline performed well, achieving 97.00% precision, 94.55% recall, and 95.76% F1 score. The developed Python script was integrated into a GUI, making it a minimal program that dentists can use in the clinic without having to deal with complex coding processes.

The degree of difficulty associated with tooth extractions can range from minimally invasive procedures to complex surgical interventions needing general anesthesia. Categorizing the difficulty of tooth extractions and calculating the time required are important for oral and maxillofacial surgeons [20]. It may also be important for some insurance systems in Asian countries [4]. According to Parant [45], easy extractions are classified as Grade I (requiring only forceps) or Grade II (requiring the osteotomy), while difficult extractions are classified as Grade III (requiring both osteotomy and coronal section) or Grade IV (involving complex procedures such as root resection). Kwon et al. [20] introduced a hybrid model that integrates a convolutional neural network (CNN) utilizing panoramic X-ray images with a multilayer perceptron (MLP) leveraging patient clinical data to estimate the extraction time for mandibular third molars. The combined CNN+MLP model achieved a strong correlation (R = 0.8315) with the test data, predicting extraction times with a mean error of 2.95 min.

In the literature, some studies detect only Pell and Gregory [29,30,31] and Winter [9,15,29,30,31,32] categories based on AI; however, articles on determining the extraction difficulty of mandibular third molars are extremely limited [33,34,35,36,37]. Yoo et al. (2021) [33] proposed a CNN-based model to predict the extraction difficulty of mandibular third molars using PRs. A total of 1053 mandibular third molars from 600 panoramic images were used and labeled according to the Pederson difficulty index by three experts. Zero-padding was applied to the images for preprocessing. The proposed method utilized a two-stage process of region-of-interest (ROI) detection and classification. For ROI detection, a Single-Shot Multibox detector-based object detection model was used and VGG16 acted as a pre-trained backbone. The detected regions were then sent to the ResNet-34-based model for the classification of Pederson difficulty scores. Accuracies of 78.91%, 82.03%, and 90.23% were achieved for C1 (depth), C2 (ramal relationship), and C3 (angulation), respectively. The RMSE value was calculated as 0.6738 when the model predicted the PDS scores. The use of only PRs was mentioned in the study as a limitation that may affect the accuracy of the model. It was emphasized that although PRs extend the range of anatomical structures in 2D, the possibility of unavoidable distortions in both vertical and horizontal dimensions, as well as transverse angulation and dilacerations were extremely difficult to assess. Lee et al. [34] (2022) developed a deep learning approach to predict the extraction difficulty of mandibular third molars from PRs and the probability of inferior alveolar nerve (IAN) injury. A total of 8720 mandibular third molars in 4903 PRs were evaluated by seven dentists. The Retinanet (ResNet-152) model was used for detection, and the Vision Transformer (R50+ViT-L/32) was used for classification. Preprocessing of images involved size adjustment, Contrast-Limited Adaptive Histogram Equalization (CLAHE), and data augmentation. In addition, the ROI was cropped to 700 × 700 pixels to contain the mandibular third molar, adjacent teeth, and IAN. The proposed method, instead of Pederson, estimated extraction difficulty in four classes: vertical eruption, soft tissue impaction, partial bone impaction, and complete bone impaction. It also assessed IAN injury risk in three levels: low, moderate, and high. For extraction difficulty estimation, the R50+ViT-L/32 model indicated the highest performance with 83.5% accuracy, 66.35% F1 score, and 92.79% AUROC, while the ResNet-34 model showed lower performance with 80.07% accuracy and 63.28% F1 score, and the ResNet-152 model showed lower performance with 82.18% accuracy and 63.23% F1 score. In the IAN injury risk classification, the proposed method gave 81.1% accuracy, 75.55% F1 score, and 90.02% AUROC. Li et al. [35] (2023) proposed a model combining deep learning and super-resolution (SR) technologies to estimate extraction difficulty. A total of 608 panoramic images obtained from two medical centers were used; the mandibular third molar, adjacent teeth, and inferior alveolar nerve regions as ROIs were cropped to 200 × 200 pixels, and then their dimensions were normalized to 800 × 800 pixels. High-resolution images were created from low-resolution images using a GAN-based method for super-resolution, and this process was performed by training the GAN with low–high-resolution pairs of images with Gaussian noise added. Feature extraction was performed with 2048 features obtained from the ResNet152 model, and statistical methods and LASSO regression were used for feature selection to determine the final features. The features were given as input to conventional machine learning models including AdaBoost, Logistic Regression, K-Nearest Neighbor, MLP, and NaiveBayes. Pederson scores were divided into two categories (≤5 and >5) and were used as output classes. However, these categories were not named “easy” or “difficult” in this study; only the scores were used for classification. In the classification performance of extraction difficulty, the Model-SR Logistic Regression machine learning algorithm showed the most optimal algorithm performance with an accuracy of 79.8%, sensitivity of 85.5%, specificity of 60.9%, and AUC of 0.779. The overall AUC of Model-SR was 0.963, which was found to be superior to expert dentists. Trachoo et al. [37] (2024) classified the extraction difficulty of impacted mandibular third molars from 1367 PRs collected retrospectively between 2021 and 2023 as novice, intermediate, and expert, taking into account the Pederson index, according to the level of dentists who can perform the surgical intervention. Images were resized to 300 × 600 pixels, and data augmentation techniques (zooming, resampling, horizontal flipping) were used. In PRs, images were split in the middle to analyze the right and left teeth separately. The system consisted of a three-stage model: ResNet101V2 performed binary classification to determine the presence of impacted lower third molars and achieved 86.71% accuracy; RetinaNet performed object detection to determine the location of LM3 and showed outstanding performance with 99.28% mAP; and Vision Transformer (ViT) performed multiclass classification to estimate surgical difficulty levels (novice, intermediate, expert) and achieved an average accuracy of 78.99%. Chindanuruks et al. (2024) [36] trained the YOLOv5x model for the Pernambuco Index with 1730 PRs taken in 2021–2023. The images were in .BMP format, labeled with LabelImg, and converted to YOLO format. As a preprocess, the images were resized to 416 × 416 pixels, and a horizontal flip was applied for data augmentation. The algorithm assessed six radiographic criteria, namely depth (level: 87% accuracy, 85.1% AUC-PRC, mAP@0.5: 87%), ramus relationship (77% accuracy, 76.8% AUC-PRC, mAP@0.5: 77%), angulation (Winter: 84% accuracy, 89.4% AUC-PRC, mAP@0.5: 84%), root number (89% accuracy, 88% AUC-PRC, mAP@0.5: 89%), root curvature (dilacerated: 61% accuracy, 72.2% AUC-PRC, mAP@0.5: 61%) and contact with the second molar (contact: 82% accuracy, 85% AUC-PRC, mAP@0.5: 82%). Surgical difficulty was classified as low, moderate, and high by adding age and body mass index (BMI). The YOLOv5x model used was the version with the highest performance in the series, the overall AUC-ROC score was found to be 85%, 77%, and 74% for low, medium, and high classes, respectively, and the model’s agreement with experts was found to be ICC = 0.802.

When the studies in the literature are examined, it can be seen that most of them perform classification after cropping the images. Additionally, preprocessing steps other than resizing are also performed. Single-shot models, such as YOLO11, perform both localization and classification by analyzing the image in one pass, consequently reducing the computational cost. Achieving high results without complex preprocessing and cropping is one of the prominent advantages of the study. PRs are complex images by nature, with anatomical structures that vary according to the person. Different anatomical structures may have various variations such as fillings, missing teeth, screws, plates, wires, artifacts, and superimposition situations. Despite these disadvantages, the third molar position was found to be accurate in almost all images. The results obtained with the pipeline proposed in this study were successful in the automatic calculation of mandibular third molar extraction difficulty, in comparison with other studies. The high precision, recall, F1 score, and mAP values obtained in this study showed that YOLO11 outperformed similar studies with high accuracy and low error rate in Winter, class, and level tasks. In this study, YOLO11, whose performance was tested for the first time in dental diagnosis processes, has an optimized structure compared to previous versions. It offers better overall performance with more parameter control and modern hyperparameter settings [40]. These features of YOLO11 make it a superior model with regard to both speed and accuracy, and it can be favored in AI-supported diagnosis tasks.

### Limitations

The fact that this study was conducted only with panoramic radiographs means that clinical findings and other imaging modalities were not integrated. Various factors influencing extraction difficulty, including anatomical challenges, root morphology, bone density, patient age, gender, mouth-opening capacity, body mass index (BMI, kg/m2), proximity to vital structures, and the surgeon’s expertise, were not comprehensively assessed. In the future, evaluating these additional parameters using three-dimensional imaging techniques (e.g., CBCT), and using larger datasets obtained from different centers may increase the accuracy and generalizability of the model. The limitations of the developed software and GUI include the possibility of performance degradation in devices with low hardware capacity due to the high computational power required by the YOLO11 model, and the fact that it cannot be generalized for other tooth types or difficulty assessment methods because it focuses only on mandibular third molars and the Pederson difficulty index.

## 5. Conclusions

This study highlights the effectiveness of YOLO11 models in predicting mandibular third molar extraction difficulty using panoramic radiographs and the Pederson difficulty index. The YOLO11 nano and medium sub-models demonstrated a strong balance between accuracy and efficiency. Their integration into a user-friendly GUI allows for rapid assessment, improving surgical planning and patient management. Future research should incorporate clinical factors and advanced imaging to enhance model accuracy and applicability further.

## Figures and Tables

**Table 1:** Criteria and scores of the Pederson index [5,41,42,43].

Criterion		Values
**Winter**	Mesioangular	1
	Horizontal/Transverse	2
	Vertical	3
	Distoangular	4
**Depth**	Level A	1
	Level B	2
	Level C	3
**Ramus**	Class 1	1
	Class 2	2
	Class 3	3
**Score**	Slightly difficult	3–4
	Moderately difficult	5–6
	Very difficult	7–10

* Pederson’s original index classified 5–7 scores as ‘Moderately Difficult’ [43].

**Table 2:** Training and testing results for YOLO11 sub-models on Winter angulation.

Model	Training								Testing						
YOLO11	Prediction	Img	Inst	P	R	F1-S	mAP@.5	mAP@.5:.95	Img	Inst	P	R	F1-S	mAP@.5	mAP@.5:.95
**nano**	Mesioangular	118	160	0.939	0.975	0.957	0.984	0.900	136	174	0.970	0.989	0.979	0.983	0.875
	Distoangular	56	62	0.962	0.809	0.879	0.946	0.812	20	26	0.851	0.876	0.863	0.933	0.791
	Horizontal	68	88	0.935	0.977	0.956	0.965	0.853	76	92	0.99	0.989	0.989	0.994	0.874
	Vertical	124	184	0.811	0.973	0.885	0.956	0.815	148	200	0.949	0.965	0.957	0.988	0.858
	**All**	280	494	0.912	0.934	0.923	0.963	0.845	280	492	0.940	0.955	0.947	0.975	0.849
**small**	Mesioangular	118	160	0.951	1.00	0.975	0.989	0.899	136	174	0.962	0.977	0.969	0.983	0.872
	Distoangular	56	62	0.946	0.742	0.832	0.946	0.836	20	26	0.682	0.769	0.723	0.810	0.685
	Horizontal	68	88	0.967	0.977	0.972	0.964	0.86	76	92	0.962	0.989	0.975	0.994	0.884
	Vertical	124	184	0.876	0.963	0.917	0.958	0.829	148	200	0.941	0.970	0.955	0.980	0.860
	**All**	280	494	0.935	0.921	0.928	0.964	0.856	280	492	0.887	0.926	0.906	0.942	0.825
**medium**	Mesioangular	118	160	0.924	0.994	0.958	0.991	0.896	136	174	0.959	0.994	0.976	0.991	0.885
	Distoangular	56	62	0.963	0.842	0.898	0.958	0.842	20	26	0.675	0.88	0.764	0.869	0.715
	Horizontal	68	88	0.965	0.941	0.953	0.956	0.854	76	92	0.989	0.978	0.983	0.990	0.876
	Vertical	124	184	0.893	0.962	0.926	0.949	0.812	148	200	0.950	0.955	0.952	0.986	0.870
	**All**	280	494	0.936	0.935	0.935	0.964	0.851	280	492	0.893	0.952	0.922	0.959	0.837
**large**	Mesioangular	118	160	0.939	0.981	0.960	0.987	0.89	136	174	0.969	1.00	0.984	0.990	0.869
	Distoangular	56	62	0.954	0.666	0.784	0.921	0.804	20	26	0.984	0.769	0.863	0.942	0.769
	Horizontal	68	88	0.927	0.977	0.951	0.972	0.868	76	92	0.951	1.00	0.975	0.995	0.880
	Vertical	124	184	0.822	0.954	0.883	0.944	0.803	148	200	0.941	0.985	0.962	0.989	0.860
	**All**	280	494	0.910	0.895	0.902	0.956	0.842	280	492	0.961	0.939	0.950	0.979	0.845
**extra large**	Mesioangular	118	160	0.949	1.00	0.974	0.993	0.916	136	174	0.967	0.983	0.975	0.990	0.880
	Distoangular	56	62	0.914	0.790	0.847	0.917	0.808	20	26	0.749	0.917	0.825	0.905	0.770
	Horizontal	68	88	0.935	0.976	0.955	0.971	0.861	76	92	0.979	0.996	0.987	0.995	0.884
	Vertical	124	184	0.834	0.973	0.898	0.947	0.809	148	200	0.934	0.984	0.958	0.990	0.870
	**All**	280	494	0.908	0.935	0.921	0.957	0.848	280	492	0.907	0.97	0.937	0.970	0.851

**Table 3:** Training and testing results for YOLO11 sub-models on Pell and Gregory.

Model	Training								Testing						
YOLO11	Prediction	Img	Inst	P	R	F1-S	mAP@.5	mAP@.5:.95	Img	Inst	P	R	F1-S	mAP@.5	mAP@.5:.95
**nano**	Class I	96	120	0.973	0.983	0.978	0.990	0.913	100	124	0.936	0.950	0.943	0.979	0.901
	Class II	226	360	0.954	0.980	0.967	0.991	0.937	200	308	0.899	0.981	0.938	0.976	0.919
	Class III	54	60	0.876	0.883	0.879	0.956	0.885	76	96	0.931	0.844	0.885	0.940	0.838
	**All**	280	540	0.934	0.949	0.941	0.979	0.911	280	528	0.922	0.925	0.923	0.965	0.886
**small**	Class I	96	120	0.959	0.992	0.975	0.995	0.917	100	124	0.910	0.977	0.942	0.974	0.895
	Class II	226	360	0.918	0.992	0.954	0.991	0.938	200	308	0.888	0.990	0.936	0.964	0.902
	Class III	54	60	0.884	0.892	0.888	0.925	0.872	76	96	0.963	0.808	0.879	0.908	0.831
	**All**	280	540	0.921	0.958	0.939	0.970	0.909	280	528	0.920	0.925	0.922	0.949	0.876
**medium**	Class I	96	120	0.973	0.992	0.982	0.995	0.927	100	124	0.871	0.984	0.924	0.983	0.913
	Class II	226	360	0.929	0.997	0.962	0.982	0.932	200	308	0.804	1.000	0.891	0.968	0.912
	Class III	54	60	0.922	0.817	0.866	0.926	0.878	76	96	0.847	0.865	0.856	0.908	0.834
	**All**	280	540	0.941	0.935	0.938	0.968	0.912	280	528	0.841	0.949	0.892	0.953	0.886
**large**	Class I	96	120	1.00	0.979	0.989	0.994	0.911	100	124	0.931	0.986	0.958	0.982	0.899
	Class II	226	360	0.938	1.00	0.968	0.993	0.936	200	308	0.861	0.990	0.921	0.979	0.919
	Class III	54	60	0.961	0.816	0.883	0.961	0.870	76	96	0.863	0.771	0.814	0.921	0.817
	**All**	280	540	0.966	0.932	0.949	0.983	0.906	280	528	0.885	0.916	0.900	0.961	0.878
**extra large**	Class I	96	120	0.957	0.983	0.970	0.992	0.907	100	124	0.886	0.992	0.936	0.956	0.882
	Class II	226	360	0.903	0.994	0.946	0.985	0.927	200	308	0.861	0.974	0.914	0.956	0.901
	Class III	54	60	0.866	0.860	0.863	0.935	0.886	76	96	0.920	0.721	0.808	0.888	0.791
	**All**	280	540	0.909	0.946	0.927	0.971	0.907	280	528	0.889	0.896	0.892	0.933	0.858
**nano**	LevelA	106	146	0.966	0.945	0.955	0.99	0.923	96	134	0.947	0.955	0.951	0.985	0.924
	LevelB	130	162	0.818	0.969	0.955	0.973	0.925	106	134	0.967	0.865	0.913	0.976	0.927
	LevelC	146	206	0.935	0.990	0.887	0.991	0.923	136	190	0.950	0.958	0.954	0.988	0.931
	**All**	280	514	0.906	0.968	0.936	0.985	0.923	280	458	0.955	0.926	0.940	0.983	0.928
**small**	LevelA	106	146	0.958	0.930	0.944	0.981	0.924	96	134	0.970	0.963	0.966	0.989	0.933
	LevelB	130	162	0.851	0.969	0.906	0.966	0.928	106	134	0.948	0.952	0.950	0.984	0.946
	LevelC	146	206	0.959	0.966	0.962	0.992	0.946	136	190	0.959	0.987	0.973	0.990	0.940
	**All**	280	514	0.922	0.955	0.938	0.980	0.933	280	458	0.959	0.967	0.963	0.988	0.940
**medium**	LevelA	106	146	0.942	0.925	0.933	0.982	0.93	96	134	0.970	0.967	0.968	0.990	0.946
	LevelB	130	162	0.833	0.956	0.890	0.96	0.925	106	134	0.955	0.940	0.947	0.985	0.953
	LevelC	146	206	0.948	0.969	0.958	0.987	0.939	136	190	0.954	0.992	0.973	0.992	0.947
	**All**	280	514	0.908	0.950	0.929	0.977	0.931	280	458	0.960	0.966	0.963	0.989	0.949
**large**	LevelA	106	146	0.934	0.971	0.952	0.98	0.923	96	134	0.963	0.975	0.969	0.991	0.951
	LevelB	130	162	0.827	0.944	0.882	0.965	0.931	106	134	0.942	0.963	0.952	0.983	0.951
	LevelC	146	206	0.944	0.981	0.962	0.981	0.937	136	190	0.974	0.970	0.972	0.985	0.937
	**All**	280	514	0.902	0.965	0.932	0.975	0.93	280	458	0.96	0.969	0.964	0.986	0.946
**extra large**	LevelA	106	146	0.934	0.963	0.948	0.976	0.915	96	134	0.93	0.970	0.950	0.977	0.931
	LevelB	130	162	0.858	0.935	0.895	0.951	0.916	106	134	0.966	0.925	0.945	0.982	0.949
	LevelC	146	206	0.939	0.961	0.950	0.984	0.938	136	190	0.984	0.983	0.983	0.987	0.937
	**All**	280	514	0.910	0.953	0.931	0.970	0.923	280	458	0.960	0.960	0.960	0.982	0.939

**Table 4:** All possible combinations and scores for the Pederson index.

Angulation	Ramus	Depth	Score	Difficulty	Angulation	Ramus	Depth	Score	Difficulty
Mesioangular (1)	Class I (1)	Level A (1)	3	Slightly	Vertical (3)	Class I (1)	Level A (1)	5	Moderately
Mesioangular (1)	Class I (1)	Level B (2)	4	Slightly	Vertical (3)	Class I (1)	Level B (2)	6	Moderately
Mesioangular (1)	Class I (1)	Level C (3)	5	Moderately	Vertical (3)	Class I (1)	Level C (3)	7	Very
Mesioangular (1)	Class II (2)	Level A (1)	4	Slightly	Vertical (3)	Class II (2)	Level A (1)	6	Moderately
Mesioangular (1)	Class II (2)	Level B (2)	5	Moderately	Vertical (3)	Class II (2)	Level B (2)	7	Very
Mesioangular (1)	Class II (2)	Level C (3)	6	Moderately	Vertical (3)	Class II (2)	Level C (3)	8	Very
Mesioangular (1)	Class III (3)	Level A (1)	5	Moderately	Vertical (3)	Class III (3)	Level A (1)	7	Very
Mesioangular (1)	Class III (3)	Level B (2)	6	Moderately	Vertical (3)	Class III (3)	Level B (2)	8	Very
Mesioangular (1)	Class III (3)	Level C (3)	7	Very	Vertical (3)	Class III (3)	Level C (3)	9	Very
Horizontal (2)	Class I (1)	Level A (1)	4	Slightly	Distoangular (4)	Class I (1)	Level A (1)	6	Moderately
Horizontal (2)	Class I (1)	Level B (2)	5	Moderately	Distoangular (4)	Class I (1)	Level B (2)	7	Very
Horizontal (2)	Class I (1)	Level C (3)	6	Moderately	Distoangular (4)	Class I (1)	Level C (3)	8	Very
Horizontal (2)	Class II (2)	Level A (1)	5	Moderately	Distoangular (4)	Class II (2)	Level A (1)	7	Very
Horizontal (2)	Class II (2)	Level B (2)	6	Moderately	Distoangular (4)	Class II (2)	Level B (2)	8	Very
Horizontal (2)	Class II (2)	Level C (3)	7	Very	Distoangular (4)	Class II (2)	Level C (3)	9	Very
Horizontal (2)	Class III (3)	Level A (1)	6	Moderately	Distoangular (4)	Class III (3)	Level A (1)	8	Very
Horizontal (2)	Class III (3)	Level B (2)	7	Very	Distoangular (4)	Class III (3)	Level B (2)	9	Very
Horizontal (2)	Class III (3)	Level C (3)	8	Very	Distoangular (4)	Class III (3)	Level C (3)	10	Very

## Data Availability

The datasets can be shared with researchers who wish to conduct studies upon reasonable request.

## Abbreviations

The following abbreviations are used in this manuscript:

AI AP	Artificial Intelligence Average Precision
AUC-PRC	Area Under the Precision-Recall Curve
AUROC	Area Under the Receiver Operating Characteristic Curve
BMI	body mass index
CBCT	cone beam computed tomography
CLAHE	Contrast-Limited Adaptive Histogram Equalization
CM	confusion matrix
CNN	convolutional neural network
FN	False Negative
FP	False Positive
F1-S	F1 score
GUI	graphical user interface
ICC	Intraclass Correlation Coefficient
IAN	inferior alveolar nerve
Img	images
Ins	instances
IoU	Intersection over Union
MLP	multilayer perceptron
mAP N/A	mean Average Precision Not Applicable
PNG	Portable Network Graphics
PRs	panoramic radiographs
P	precision
R	recall
ROI	region of interest
SR	super-resolution
TP	True Positive
ViT	Vision Transformer
YOLO	You Only Look Once

## References

[1] Albayati M.T., Bede S.Y. (2023). Reliability of two difficulty indexes in predicting the surgical extraction difficulty of impacted mandibular third molars. J. Oral Med. Oral Surg..

[2] Kim J.Y., Yong H.S., Park K.H., Huh J.K. (2019). Modified difficult index adding extremely difficult for fully impacted mandibular third molar extraction. J. Korean Assoc. Oral Maxillofac. Surg..

[3] Bali A., Bali D., Sharma A., Verma G. (2013). Is Pederson Index a True Predictive Difficulty Index for Impacted Mandibular Third Molar Surgery? A Meta-analysis. J. Maxillofac. Oral Surg..

[4] Njokanma A.R., Aborisade A., Kuye O.F., Amedari M.I., Njokanma A.H. (2024). Does Pederson Difficulty Index Accurately Predict the Difficulty of Mandibular Third Molar Extraction?. Niger. J. Basic Clin. Sci..

[5] Pederson G.W., Pederson G.W. (1988). Surgical removal of tooth. Oral Surgery.

[6] Pell G.J. (1933). Impacted mandibular third molars, classification and modified technique for removal. Dent. Dig..

[7] Winter G.B. (1926). Principles of Exodontia as Applied to the Impacted Mandibular Third Molar.

[8] Atieh M.A. (2010). Diagnostic accuracy of panoramic radiography in determining relationship between inferior alveolar nerve and mandibular third molar. J. Oral Maxillofac. Surg..

[9] Zirek T., Öziç M.Ü., Tassoker M. (2024). AI-Driven localization of all impacted teeth and prediction of winter angulation for third molars on panoramic radiographs: Clinical user interface design. Comput. Biol. Med..

[10] Erturk M., Öziç M.Ü., Tassoker M. (2024). Deep Convolutional Neural Network for Automated Staging of Periodontal Bone Loss Severity on Bite-wing Radiographs: An Eigen-CAM Explainability Mapping Approach. J. Imaging Inform. Med..

[11] Karakuş R., Öziç M.Ü., Tassoker M. (2024). AI-Assisted Detection of Interproximal, Occlusal, and Secondary Caries on Bite-Wing Radiographs: A Single-Shot Deep Learning Approach. J. Imaging Inform. Med..

[12] Park C., Took C.C., Seong J.-K. (2018). Machine learning in biomedical engineering. Biomed. Eng. Lett..

[13] Pouyanfar S., Sadiq S., Yan Y., Tian H., Tao Y., Reyes M.P., Shyu M.L., Chen S.C., Iyengar S.S. (2018). A survey on deep learning: Algorithms, techniques, and applications. ACM Comput. Surv..

[14] Vranckx M., Van Gerven A., Willems H., Vandemeulebroucke A., Ferreira Leite A., Politis C., Jacobs R. (2020). Artificial intelligence (AI)-driven molar angulation measurements to predict third molar eruption on panoramic radiographs. Int. J. Environ. Res. Public Health.

[15] Celik M.E. (2022). Deep learning based detection tool for impacted mandibular third molar teeth. Diagnostics.

[16] Chen S.-L., Chou H.-S., Chuo Y., Lin Y.-J., Tsai T.-H., Peng C.-H., Tseng A.-Y., Li K.-C., Chen C.-A., Chen T.-Y. (2024). Classification of the Relative Position between the Third Molar and the Inferior Alveolar Nerve Using a Convolutional Neural Network Based on Transfer Learning. Electronics.

[17] Vinayahalingam S., Xi T., Bergé S., Maal T., De Jong G. (2019). Automated detection of third molars and mandibular nerve by deep learning. Sci. Rep..

[18] Banar N., Bertels J., Laurent F., Boedi R.M., De Tobel J., Thevissen P., Vandermeulen D. (2020). Towards fully automated third molar development staging in panoramic radiographs. Int. J. Leg. Med..

[19] De Tobel J., Radesh P., Vandermeulen D., Thevissen P.W. (2017). An automated technique to stage lower third molar development on panoramic radiographs for age estimation: A pilot study. J. Forensic Odonto-Stomatol..

[20] Kwon D., Ahn J., Kim C.-S., Kang D.O., Paeng J.-Y. (2022). A deep learning model based on concatenation approach to predict the time to extract a mandibular third molar tooth. BMC Oral Health.

[21] Milani O.H., Atici S.F., Allareddy V., Ramachandran V., Ansari R., Cetin A.E., Elnagar M.H. (2024). A fully automated classification of third molar development stages using deep learning. Sci. Rep..

[22] Kempers S., van Lierop P., Hsu T.-M.H., Moin D.A., Bergé S., Ghaeminia H., Xi T., Vinayahalingam S. (2023). Positional assessment of lower third molar and mandibular canal using explainable artificial intelligence. J. Dent..

[23] Ariji Y., Mori M., Fukuda M., Katsumata A., Ariji E. (2022). Automatic visualization of the mandibular canal in relation to an impacted mandibular third molar on panoramic radiographs using deep learning segmentation and transfer learning techniques. Oral Surg. Oral Med. Oral Pathol. Oral Radiol..

[24] Kim J.-Y., Kahm S.H., Yoo S., Bae S.-M., Kang J.-E., Lee S.H. (2023). The efficacy of supervised learning and semi-supervised learning in diagnosis of impacted third molar on panoramic radiographs through artificial intelligence model. Dentomaxillofacial Radiol..

[25] Joo Y., Moon S.-Y., Choi C. (2023). Classification of the relationship between mandibular third molar and inferior alveolar nerve based on generated mask images. IEEE Access.

[26] Zhu T., Chen D., Wu F., Zhu F., Zhu H. (2021). Artificial intelligence model to detect real contact relationship between mandibular third molars and inferior alveolar nerve based on panoramic radiographs. Diagnostics.

[27] Lo Casto A., Spartivento G., Benfante V., Di Raimondo R., Ali M., Di Raimondo D., Tuttolomondo A., Stefano A., Yezzi A., Comelli A. (2023). Artificial intelligence for classifying the relationship between impacted third molar and mandibular canal on panoramic radiographs. Life.

[28] Takebe K., Imai T., Kubota S., Nishimoto A., Amekawa S., Uzawa N. (2023). Deep learning model for the automated evaluation of contact between the lower third molar and inferior alveolar nerve on panoramic radiography. J. Dent. Sci..

[29] Maruta N., Morita K.-I., Harazono Y., Anzai E., Akaike Y., Yamazaki K., Tonouchi E., Yoda T. (2023). Automatic machine learning-based classification of mandibular third molar impaction status. J. Oral Maxillofac. Surg. Med. Pathol..

[30] Sukegawa S., Matsuyama T., Tanaka F., Hara T., Yoshii K., Yamashita K., Nakano K., Takabatake K., Kawai H., Nagatsuka H. (2022). Evaluation of multi-task learning in deep learning-based positioning classification of mandibular third molars. Sci. Rep..

[31] Aravena H., Arredondo M., Fuentes C., Taramasco C., Alcocer D., Gatica G. (2023). Predictive Treatment of Third Molars Using Panoramic Radiographs and Machine Learning. Proceedings of the 2023 19th International Conference on Wireless and Mobile Computing, Networking and Communications (WiMob).

[32] Lei Y., Chen X., Wang Y., Tang R., Zhang B. (2023). A Lightweight Knowledge-Distillation-Based Model for the Detection and Classification of Impacted Mandibular Third Molars. Appl. Sci..

[33] Yoo J.-H., Yeom H.-G., Shin W., Yun J.P., Lee J.H., Jeong S.H., Lim H.J., Lee J., Kim B.C. (2021). Deep learning based prediction of extraction difficulty for mandibular third molars. Sci. Rep..

[34] Lee J., Park J., Moon S.Y., Lee K. (2022). Automated Prediction of Extraction Difficulty and Inferior Alveolar Nerve Injury for Mandibular Third Molar Using a Deep Neural Network. Appl. Sci..

[35] Li W., Li Y., Liu X. (2023). Transfer learning-based super-resolution in panoramic models for predicting mandibular third molar extraction difficulty: A multi-center study. Med. Data Min..

[36] Chindanuruks T., Jindanil T., Cumpim C., Sinpitaksakul P., Arunjaroensuk S., Mattheos N., Pimkhaokham A. (2024). Development and validation of a deep learning algorithm for the classification of the level of surgical difficulty in impacted mandibular third molar surgery. Int. J. Oral Maxillofac. Surg..

[37] Trachoo V., Taetragool U., Pianchoopat P., Sukitporn-udom C., Morakrant N., Warin K. (2024). Deep Learning for Predicting the Difficulty Level of Removing the Impacted Mandibular Third Molar. Int. Dent. J..

[38] Adamska P., Adamski Ł.J., Musiał D., Tylek K., Studniarek M., Wychowańński P., Kaczoruk-Wieremczuk M., Pyrzowska D., Jereczek-Fossa B.A., Starzyńska A. (2020). Panoramic radiograph–a useful tool to assess the difficulty in extraction of third molars. Eur. J. Transl. Clin. Med..

[39] Ansari M.A.M.F., Mutha A. (2020). Digital Assessment of Difficulty in Impacted Mandibular Third Molar Extraction. J. Maxillofac. Oral Surg..

[40] Khanam R., Hussain M. (2024). YOLOv11: An Overview of the Key Architectural Enhancements. arXiv.

[41] Gbotolorun O.M., Arotiba G.T., Ladeinde A.L. (2007). Assessment of factors associated with surgical difficulty in impacted mandibular third molar extraction. J. Oral Maxillofac. Surg..

[42] Prerana G., Tantry D., Sougata K., Sivalanka S.C.S. (2021). Incidence of complications after the surgical removal of impacted mandibular third molars: A single center retrospective study. J. Acad. Dent. Educ..

[43] Yuasa H., Kawai T., Sugiura M. (2002). Classification of surgical difficulty in extracting impacted third molars. Br. J. Oral Maxillofac. Surg..

[44] Kharma M.Y., Sakka S., Aws G., Tarakji B., Nassani M.Z. (2014). Reliability of Pederson scale in surgical extraction of impacted lower third molars: Proposal of new scale. J. Oral Dis..

[45] Parant M. (1963). Petite Chirurgie de la Bouche.

